# Hypoallergenic animals: A promise of hope for allergic patients? 

**DOI:** 10.5414/ALX02454E

**Published:** 2024-03-21

**Authors:** Christiane Hilger, Bente Janssen-Weets, Kyra Swiontek

**Affiliations:** Department of Infection and Immunity, Luxembourg Institute of Health, Esch-sur Alzette, Luxembourg

**Keywords:** allergen, breed, cat, dog, horse, hypoallergenic, pet

## Abstract

Furry pets are beloved companion animals; horse riding is a popular leisure activity. So-called hypoallergenic animals have gained high interest as sensitization to animal dander and allergy to furry animals are widespread. Allergen immunotherapy to furry animals is still limited, and allergen avoidance in addition to symptomatic pharmaceutical treatment is often the only available option. Patients with an existing allergy to furry animals or with an atopic background are seeking for a hypoallergenic alternative. This review summarizes current knowledge and discusses future strategies.

## Introduction 

Furry animals are an important source of indoor allergens, leading to high sensitization rates and the risk to develop an IgE-mediated allergic disease such as allergic rhinitis or asthma. In the general adult population, sensitization rates of 10 – 14% were found in Europe and the United States [1, 2, 3, 4]. In sensitized individuals, exposure to high levels of animal allergens was associated with an increased prevalence of asthma and asthma morbidity [1]. Animal allergens are present on fur, in saliva and urine, and they are easily dispersed into the indoor environment and readily detectable in household dust. By sticking to human clothes and hair, they are transported to public places by animal owners and can elicit symptoms in sensitized individuals [5, 6, 7]. 

Pets are very popular. Recent surveys estimate that ~ 46% of the European households own a pet, cats being the most common, followed by dogs, pet birds, and small mammals [8]. In Germany, the first three rankings are occupied by furry pets: 24% of the households have a cat, 21% a dog, and 5% a small mammal [9]. Pets are even more prevalent in homes with children. They provide companionship, but they also have other physiological and psychological health benefits such as promoting physical exercise, reducing stress, and alleviating loneliness. For patients allergic to a furry animal, it is therefore tempting to look for a hypoallergenic animal. Specific cat, dog, or horse breeds are often advertised as hypoallergenic, and patients are seeking advice from clinicians. In this review, we summarize the current state of the art on hypoallergenic animals and discuss the different studies. 

## Allergens of furry animals 

Animal dander extracts are used either as skin test solutions or in in vitro IgE-binding assays to diagnose sensitization to a furry animal. Although they have in general a good diagnostic sensitivity, they contain a number of cross-reactive molecules which leads to a low specificity [10]. The identification of major and minor animal allergens and their use in clinical studies was a major breakthrough in understanding IgE sensitization profiles and their clinical relevance [11]. The most commercially available allergens are of cat or dog origin, whereas single allergens from other animal species are either not yet available or not available on all diagnostic platforms. The majority of known animal allergens belongs to either the lipocalin or the serum albumin protein families (Table 1). The secretoglobin family so far has only two allergen members, of which Fel d 1 is the major cat allergen and Ory c 3 a major allergen of rabbit. 

### Cat 

Among known cat allergens, Fel d 1 is the most important one and a highly predictive marker of cat allergy [11]. An IgE prevalence of more than 90% has been reported in cat-allergic patients, making Fel d 1 an ideal target for strategies aiming at introducing a hypoallergenic cat. The first cat that was claimed to be hypoallergenic through selective breeding was a cat marketed by Allerca. Although the company did not claim that their cat was completely free of Fel d 1, they stated that they had a very high success rate among cat-allergic clients. However, these claims have never been supported by scientific evidence, and the company finally ceased their activities [12]. There is individual variation in Fel d 1 production, and on average, unneutered males produce more Fel d 1 than females. To date, there is no scientific evidence for a hypoallergenic cat breed, even hairless Sphynx cats produce Fel d 1 [13, 14]. 

More recent approaches target blocking Fel d 1 in cats by either vaccinating cats using Fel d 1 or by adding a chicken anti-Fel d 1 IgY antibody to cat food. Both approaches aim at neutralizing Fel d 1 and avoiding human exposure to the allergen. The first approach using recombinant Fel d 1 conjugated to a virus-like particle carrying a tetanus toxin-derived T-cell epitope successfully demonstrated the production of high-affinity anti-Fel d 1 antibodies in the cat and a reduction of Fel d 1 levels in cat tears [15]. The second approach aimed at neutralizing Fel d 1 upon secretion into saliva. Polyclonal avian yolk-derived anti-Fel d 1 IgY antibodies were produced by immunizing hens, and the antibodies were added to cat food. Immunologically active Fel d 1 levels were significantly reduced in saliva and hair after a few weeks of treatment [16]. These preliminary findings have to be confirmed in larger studies, exposing cat-allergic patients with different severity scores to treated cats and controls. A third strategy aims at creating a Fel d 1 knockout cat using CRISP-Cas9 technology [17]. 

All current research approaches are targeting only Fel d 1. However the majority of clinically allergic patients are polysensitized to several cat allergens, and type-2 inflammation in young asthmatics was associated with co-sensitization to the lipocalin Fel d 4 [18]. It is not known how a cat producing reduced Fel d 1 levels would impact clinical symptoms of a polysensitized patient. 

### Dog 

IgE sensitization profiles to dogs are more complex than those to cat as there is no dominant allergen such as Fel d 1. Most children and adults are co-sensitized to more than one allergen [11]. Co-sensitization to several allergens, in particular dog lipocalins Can f 4 and Can f 6, were shown to be associated with clinical allergy in children, whereas monosensitization to Can f 5 was associated with a negative nasal provocation test (NPT) [19]. Can f 5 is present in urine of male dogs, and monosensitization to Can f 5 was shown to be a marker of sensitization to male dogs. Children monosensitized to Can f 5 tolerated a conjunctival challenge with female dog extract, but not with male extract, suggesting tolerance to female dogs [20]. 

The complex IgE sensitization profiles hamper the design of a targeted strategy to reduce dog allergen secretion. Earlier studies analyzing allergen content of dog hair of different breeds used Can f 1 as a marker for allergen content as this was the only available allergen detection assay [21, 22]. Within the same breed, there was a large individual variability, and males produced more Can f 1 than females [22]. The analysis of allergen levels measured at homes of 190 families with a dog did not show any evidence for a reduced shedding of dog allergens by dogs claimed to be hypoallergenic [21]. In another study, Can f 1 levels were quantified in hair and fur samples from so-called “hypoallergenic” dogs and”non-hypoallergenic” dogs, as well as in settled dust samples from the dogs’ homes. Hypoallergenic dogs analyzed were Labradoodles, Labrador retrievers, Poodles, Spanish Waterdogs, and Airdale terriers. They were compared to a heterogenous control group of 47 different non-hypoallergenic breeds and cross-breeds. The variability within the breeds was very high, whereas differences between breeds were small. Although even higher Can f 1 levels were detected on hair and fur of hypoallergenic dogs, these were not reflected in the environmental samples. The study did not show any evidence for a reduced allergen shedding of allergens into the environment by “hypoallergenic” dogs [23]. To date, there is no scientific evidence for the existence of a hypoallergenic dog. 

### Horse 

Data on primary horse allergy are scarce, and so far only four respiratory allergens have been characterized. Equ c 1 is a lipocalin and a major horse allergen highly predictive of allergy to horse, but it is also cross-reactive with cat Fel d 4 and dog Can f 6 [11]. Equ c 6 is a food allergen present in horse milk [11]. As horse allergy is less investigated than allergy to cat or dog, it is likely that not all allergens have yet been identified. Treatment of horse allergy is limited to avoidance or drug therapy, the efficacy of allergen-specific therapy for horse allergy being still insufficient [24]. Among horse breeds, the American Bashkir Curly Horse (or short: Curly Horse) has been promoted as hypoallergenic and suitable for horse-allergic riders. In a study with 40 horse-allergic patients, the majority showed no major decrease of nose and lung function after riding or horse brushing, and in some participants, a loss of reactivity was confirmed after 3 years [25]. In several cases however, a decrease in peak expiratory flow of more than 20% was recorded and not all individuals participated in all visits over the time of the study. It has been hypothesized that Curly Horses may produce less allergens or different proteins. 

**Figure 1.**
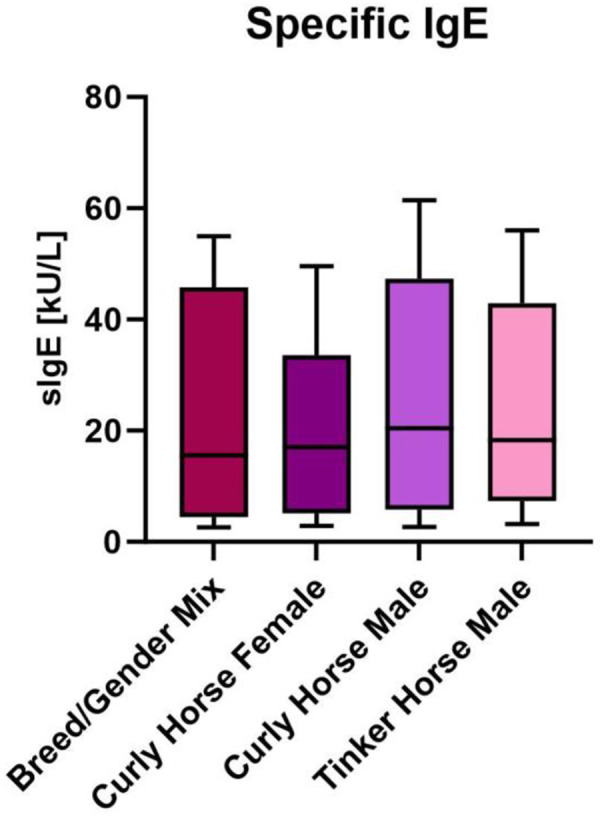
Specific IgE to horse dander was measured by ELISA in 10 patient sera and reveals no significant differences between breeds or gender. Box is delimited by interquartile range, separated by median line; whiskers extend from minimum to maximum values.

Levels of Equ c 4 were analyzed in 10 horse breeds, including Curly Horse. Equ c 4 was present in saliva and dander samples from all breeds, and levels were significantly higher in stallions than in geldings or mares. There was high individual variability of Equ c 4 levels within breeds [26]. A subsequent study analyzing levels of Equ c 1, Equ c 2, and Equ c 4 in the same breeds concluded that neither the American Curly Horse nor the Russian Bashkir Horse were associated with lower allergen levels than other horse breeds [27]. Another study investigating 32 different horse breeds found even higher allergen levels in Curly Horses, despite a very high individual variation within each breed [28]. Allergen load measured in personal nasal filters worn during grooming of Curly and Quarter Horses did not reveal any differences in allergen levels in airborne samples captured on the filters. As those studies relied on immunoassays, modified allergen sequences as well as so far unidentified proteins responsible for the claimed hypoallergenicity might have been undetected. A recent comparative proteomic analysis of the American Curly Horse, the American Quarter Horse, and a breed/gender mix of hair extracts did not find any molecular evidence supporting a hypoallergenicity of the Curly Horse [29]. Specific IgE to horse dander were measured in sera of 10 patients and showed no differences between breeds or gender (Figure 1). Although two new Equ c 1 variants were identified in all breeds, they showed a similar IgE-binding capacity as the previously characterized allergen Equ c 1.0101 and they were present in all breeds. 

A clinical study enrolling 41 patients with horse allergy to a tolerance induction protocol including regular riding 1 of 4 Curly Horses (treatment group, n = 27) found that 12 out of 12 participants of the treatment group who completed the study had a negative NPT [30]. However, a total of 15 participants in the treatment group withdraw before the end of the study and 12 of those had a positive NPT. An important drawback of the study is that it only included a control group of patients with low exposure to Curly Horse (n = 14; < 7 riding hours/year), but no control group exposed to non-Curly horse riding. Notably, patients who had longer periods of riding (> 20 hours) and whose symptoms disappeared, initially had mild symptoms [30]. In order to provide scientific evidence of the Curly Horse to be hypoallergenic, a broader clinical study involving a treatment group and a control group riding non-Curly horses has to be designed, including a categorization by symptom scores and IgE profiling of patients as well as a determination of allergen content of the horses those patients are exposed to. Individual variations in allergen content are an important factor in assessing tolerance to individual horses. It is not excluded that tolerance could be induced in mildly allergic patients regardless of the horse breed, analogous to tolerance induction protocols in food allergy. 

### Other furry animals 

Bos d 2, the major respiratory allergen in cattle, was quantified in hair samples from 16 different cattle breeds. Allergen levels varied greatly between individual animals, but levels were independent from the cattle breed or gender [31]. 

So far, there are no studies on allergen content of different breeds of small mammals such as rabbit, guinea pig, or ferret. Several species of hamsters are available as pets and laboratory animals. Although none of them is reported as hypoallergenic, the major allergens from Golden and European hamster differ from those of the *Phodopus* species to which the Siberian and Roborovski dwarf hamsters belong. As Mes a 1 and Phod s 1 are not IgE-cross-reactive, patients allergic to Siberian hamster did not react to skin prick test solutions of European or Golden hamster [32]. But there are no reports so far of a patient allergic to one species being tolerant to the other. 

## Conclusion 

Molecular analyses of animal dander from different breeds show high individual variation in allergen levels, but do not provide any scientific evidence for the concept of hypoallergenic cats, dogs, or horses. Targeted blocking of Fel d 1, the major cat allergen, showed promising preliminary results but needs further investigation in larger clinical trials with cat-allergic patients with different degrees of symptom severity and IgE sensitization profiles. 

## Funding 

Supported by institutional funding provided by the Ministry of Higher Education and Research, Luxembourg. 

## Conflict of interest 

The authors declare no conflict of interest. 

**Table 1.** Furry-pet allergens recognized by the WHO/IUIS Allergen Nomenclature Sub-Committee.

**Allergen source**	**Biochemical family**	**Allergen**
Cat *Felis domesticus*	Secretoglobin	Fel d 1
	Lipocalin	Fel d 4, Fel d 7
	Serum albumin	Fel d 2
	Cystatin	Fel d 3
	Immunoglobulin	Fel d 5, Fel d 6
	Latherin	Fel d 8
Dog *Canis familiaris*	Lipocalin	Can f 1, Can f 2, Can f 4, Can f 6
	Serum albumin	Can f 3
	Kallikrein	Can f 5
	Niemann Pick type C2	Can f 7
	Cystatin	Can f 8
Guinea-pig *Cavia porcellus*	Lipocalin	Cav p 1, Cav p 2, Cav p 3, Cav p 6,
	Serum albumin	Cav p 4
Horse *Equus caballus*	Lipocalin	Equ c 1, Equ c 2
	Serum albumin	Equ c 3
	Latherin	Equ c 4
	Lysozyme	Equ c 6
Hamster (Golden) *Mesocricetus auratus*	Lipocalin	Mes a 1
Hamster (Siberian) *Phodopus sungorus*	Lipocalin	Phod s 1
Mouse *Mus musculus*	Lipocalin	Mus m 1
Rabbit *Oryctolagus cuniculus*	Lipocalin	Ory c 1, Ory c 2, Ory c 4
	Secretoglobin	Ory c 3
Rat *Rattus norvegicus*	Lipocalin	Rat n 1
